# Minimal residual disease detection by mutation-specific droplet digital PCR for leukemia/lymphoma

**DOI:** 10.1007/s12185-023-03566-2

**Published:** 2023-03-03

**Authors:** Ryota Shirai, Tomoo Osumi, Dai Keino, Kazuhiko Nakabayashi, Toru Uchiyama, Masahiro Sekiguchi, Mitsuteru Hiwatari, Masanori Yoshida, Kaoru Yoshida, Yuji Yamada, Daisuke Tomizawa, Seido Takae, Nobutaka Kiyokawa, Kimikazu Matsumoto, Takako Yoshioka, Kenichiro Hata, Toshinori Hori, Nao Suzuki, Motohiro Kato

**Affiliations:** 1 Department of Pediatric Hematology and Oncology Research, National Research Institute for Child Health and Development, Tokyo, Japan; 2 Department of Pediatrics, Yokohama City University Graduate School of Medicine, Kanagawa, Japan; 3 Children’s Cancer Center, National Center for Child Health and Development, Tokyo, Japan; 4 Department of Pediatrics, St. Marianna University School of Medicine Hospital, Kawasaki, Japan; 5 Division of Hematology/Oncology, Kanagawa Children’s Medical Center, Yokohama, Japan; 6 Department of Maternal-Fetal Biology, National Research Institute for Child Health and Development, Tokyo, Japan; 7 Department of Human Genetics, National Research Institute for Child Health and Development, Tokyo, Japan; 8 Department of Pediatrics, the University of Tokyo, 7-3-1 Hongo, Bunkyo-Ku, Tokyo, 113-8655 Japan; 9 Department of Obstetrics and Gynecology, St. Marianna University School of Medicine, Kanagawa, Japan; 10 Department of Pathology, National Center for Child Health and Development, Tokyo, Japan; 11 Department of Pediatrics, Aichi Medical University, Nagakute, Japan; 12 Department of Human Molecular Genetics, Gunma University Graduate School of Medicine, Gunma, Japan; 13 Department of Pediatrics, School of Medicine, Teikyo University, Tokyo, Japan

**Keywords:** Droplet digital PCR, Minimal residual disease, Acute lymphoblastic leukemia, Single nucleotide variant, Cryopreserved ovarian tissue

## Abstract

Minimal residual disease (MRD) is usually defined as the small number of cancer cells that remain in the body after treatment. The clinical significance of MRD kinetics is well recognized in treatment of hematologic malignancies, particularly acute lymphoblastic leukemia (ALL). Real time quantitative PCR targeting immunoglobulin (Ig) or T-cell receptor (TCR) rearrangement (PCR-MRD), as well as multiparametric flow cytometric analysis targeting antigen expression, are widely used in MRD detection. In this study, we devised an alternative method to detect MRD using droplet digital PCR (ddPCR), targeting somatic single nucleotide variants (SNVs). This ddPCR-based method (ddPCR-MRD) had sensitivity up to 1E-4. We assessed ddPCR-MRD at 26 time points from eight T-ALL patients, and compared it to the results of PCR-MRD. Almost all results were concordant between the two methods, but ddPCR-MRD detected micro-residual disease that was missed by PCR-MRD in one patient. We also measured MRD in stored ovarian tissue of four pediatric cancer patients, and detected 1E-2 of submicroscopic infiltration. Considering the universality of ddPCR-MRD, the methods can be used as a complement for not only ALL, but also other malignant diseases regardless of tumor-specific Ig/TCR or surface antigen patterns.

**Supplementary Information:**

The online version contains supplementary material available at 10.1007/s12185-023-03566-2.

## Introduction

Minimal residual disease (MRD) is generally defined as residual malignant cells under the range of microscopic detection. Numerous studies demonstrated that MRD kinetics during chemotherapy is strongly associated with relapse risk of the malignant diseases, and quantitative assessment of MRD has been widely used as the most potent prognostic factor, particularly in acute lymphoblastic leukemia (ALL) [1–3] and acute myeloid leukemia [4, 5]. The concept of MRD as a prognostic factor is also applied to other malignant diseases, such as malignant lymphoma [6–8], and solid tumors [9, 10]. 

Multiparametric flow cytometry (FCM) and real time quantitative PCR (qPCR) are widely recognized as standard methods to measure MRD in ALL [11, 12]. However, each method has its own challenges. Multiparametric flow cytometry analysis of leukemia-associated immunophenotypes (FCM-MRD) requires fresh viable cells and the detection threshold is usually 1E-4 in 4 to 6 colors and deeper in more than 6 colors [13, 14]. On the other hand, real-time quantitative polymerase chain reaction of T-cell receptor (TCR)/immunoglobulin (Ig) gene (PCR-MRD) requires specific molecular targets, such as Ig/TCR rearrangements [13], and therefore, PCR-MRD cannot be used for some patients, particularly those with acute myeloid leukemia (AML) because Ig/TCR rearrangements in AML are immature and unusual [15, 16].

An advanced method using droplet digital PCR (ddPCR) that performs absolute quantification could overcome these limitations and potentially provide more universal and sensitive MRD analysis as well as next generation sequencing (NGS). Compared to conventional real-time quantitative PCR-MRD, ddPCR-MRD can be more sensitive method and has the potential to detect clonal evolution, without requirement primers for individual patients and standard curves for each assay [16–22]. Della Starza et al. compared ddPCR to qPCR for Ig/TCR gene arrangement detection in 23 ALL patients demonstrating that ddPCR had essential advantages such as greater applicability, greater sensitivity, and lower time consuming [23]. In recent years, some reports explored ddPCR based MRD targeting single nucleotide variants (SNVs) or translocation in leukemia like *IDH2* variant in AML, *NT5C2* and *PRPS1* variant in ALL, *BCR*-*ABL1* in ALL [24–27].

We devised a new ddPCR based MRD assay targeting tumor-specific SNVs (ddPCR-MRD) and compared it with PCR-MRD in patients with T-cell ALL (T-ALL). We also applied the method to detection of residual infiltration into stored ovarian tissues, collected for fertility preservation.

## Methods

### Patients and samples

This study was approved by the ethics committee the National Center for Child Health and Development (NCCHD) (#1035), and the required written informed consent was obtained from parents or guardians. First, eight patients with T-ALL undergoing continuous PCR-MRD evaluation from 2004 to 2018 at the University of Tokyo Hospital and NCCHD were retrospectively selected. MRD in the bone marrow was also assessed by ddPCR-MRD at the same follow-up time points as PCR-MRD and the results of MRD from the two methods were compared. Follow-up points were several time points during chemotherapy, including the end of induction and end of the first consolidation.

### Ovarian tissue cryopreservation (OTC)

Four pediatric cancer patients who had their ovarian tissues cryopreserved for fertility preservation during bone marrow remission at the NCCHD and St. Marianna University School of Medicine between 2016 and 2018 were also enrolled to assess MRD contamination in ovarian tissues using the new ddPCR analysis (Table 1). The bone marrow was in remission at the time of OTC in all patients. The detailed methods of ovarian tissue cryopreservation have been described in previous papers [28, 29]. Briefly, the medulla was removed from biopsy specimens taken at the time of ovarian tissue cryopreservation. Thin layers of ovarian cortices (1–2 mm thickness) were prepared and cut into small strips (0.5–1 × 0.5–1 cm, 1–2 mm thickness) and vitrified in the CryoSupport device. The edges of thin layers of ovarian cortices were used to confirm the absence of malignant cells. The medullas were frozen and preserved in the extracellular cryoprotectant CP-1 (Kyokuto Pharmaceutical Industrial, Tokyo, Japan) at − 80 °C until use and used for MRD evaluation in three out of four cases. MRD evaluations of unique patient number (UPN) 2 and UPN4 were performed on multiple randomly selected medulla sites (Table 1). For UPN1, MRD evaluation was performed using formalin-fixed paraffin-embedded (FFPE) ovarian cortical tissues.

**Table 1:** Clinical characteristics and results of ovarian ddPCR MRD

UPN	Age at OTC (Age at diagnosis)	Diagnosis	Ovarian tissue histology	Ovarian ddPCR MRD (%)	Ovary sample	Period from the start of treatment to OTC (months)	Follow-up period after OTC	Status
1	11y2m (10y8m)	B-cell precursor acute lymphoblastic leukemia	Negative	0.21	FFPE (1 piece)	6	3y1m	Alive
2	10y9m (10y5m)	Acute myeloid leukemia	Negative	Negative	Frozen (9 pieces)	4	1y8m	Alive
3	11y11m (11y9m)	Intracranial undifferentiated pleomorphic sarcoma	Negative	Negative	Frozen (1 piece)	2	1y9m	Alive
4	16y11m (15y9m)	γδT-cell lymphoma	Negative	Negative	Frozen (9 pieces)	14	1y4m	Alive

*UPN* unique patient number; *OTC* ovarian tissue cryopreservation; *ddPCR* droplet digital PCR; *MRD* minimal residual disease; *HR* high risk; *y* years; *m* months

### Histological analyses and IHC

Pieces of ovarian cortex were fixed in 4% formaldehyde and embedded in paraffin. These were serially sectioned at a thickness of 4 µm and underwent histological evaluation by expert pathologists to confirm pathological absence of malignant cells. Residual follicles in ovarian cortices were also confirmed in all patients using a previously described method [29].

### Whole-exome sequencing

To identify tumor-specific SNVs for each patient, we performed whole-exome sequencing (WES) using DNA extracted from tumor specimens and paired blood samples at remission. Genomic DNA was extracted using the QIAamp DNA Mini kit (Qiagen, Hilden, Germany) or GeneRead DNA FFPE kit (Qiagen, Hilden, Germany). After fragmentation of the DNA to approximately 200 bp with advanced focused acoustics (Covaris, Woburn, MA), library construction was performed with a combination of the SureSelect HumanAll Exon Kit (Agilent Technology, Santa Clara, CA) and KAPA Hyper Prep Kit (Roche Diagnostics, Basel, Switzerland) according to the manufacture’s protocol. Enriched fragment libraries were then sequenced on an Illumina HiSeq 2500 in 101-bp paired-end mode. A robust bioinformatics pipeline validated previously was used for alignment and variant calling.

Short reads obtained from the sequencer were processed, mapped, and analyzed as previously described [30]. In brief, the paired-end reads were first trimmed by removing library adapters and low-quality bases at ends and then aligned to the hs37d5 sequence (GRCh37 and decoy sequences). Uniquely mapped read pairs were selected to make Sequence Alignment Map and Binary Alignment Map files, followed by removal of PCR duplicates, local realignment, and recalibration of map quality scores. Multi-sample calling with diagnostic and remission samples was employed for detection of mutations occurring in the tumor cells. Annotations of altered sites were made using the ANNOVAR software based on GRCh37 with annotation databases, including SIFT [31], Polyphen2 [32], and CADD [33].

### MRD detection by real-time quantitative PCR

MRD for all T-ALL patients was measured by qPCR using rearrangements of Ig/TCR as described previously [34, 35] at Aichi Medical University, which is a certified laboratory of EuroMRD (http://www.euromrd.org) [3, 36].

Data were analyzed according to the EuroMRD guidelines [37]. Amplification below the quantitative range of the standard curve was defined as below the quantitative range (BQR) [38].

### MRD detection by droplet digital PCR

ddPCR was used to accurately quantify allele frequency of tumor-specific SNVs detected by WES. The percentage of tumor cells was estimated from the variant allele frequencies that were calculated as ddPCR-MRD. ddPCR experiments were performed using the QX-200 Droplet Digital PCR system (Bio-Rad, Hercules, CA) according to the manufacturer’s instructions. For all assembled 22 µL polymerase chain reaction mixtures, we included ddPCR Supermix for Probes (No dUTP; Bio-Rad, Hercules, CA), primers at a final concentration of 0.25 µM, probes at a final concentration of 1 µM, and 10 units of restriction enzyme Hind III. We basically loaded 150 ng of genomic DNA per well unless the sample amount was insufficient. Each assay was performed in triplicate. Thermal cycling conditions were 95 ºC for 10 min (1 cycle), 94 °C for 30 s (ramp rate 2 °C/second, 40 cycles), the appropriate annealing temperature of each primer/probe set for 1 min (ramp rate 2 °C /second, 40 cycles), 98 °C for 10 min (1 cycle), and 4 °C hold. The emulsion was obtained using a QX-200 Droplet Generator and read by a QX-200 droplet reader, and the analysis of experimental results was performed using QuantaSoft analysis software version 1.7.4 (Bio-Rad, Hercules, CA). Each experiment included a positive and a negative control sample.

We firstly selected five to seven tumor-specific SNVs with high allele frequency in WES per each patient since even the driver gene can be lost. Secondly, among them, four primer/probe sets were selected for which the estimated melting temperature of the full-match probe and the mismatched probe of the SNV- containing sequences were greater than 5 degrees of each other. Those primer/probe sets were designed to contain FAM probe for the mutant allele and HEX probe for the wild type allele targeting each SNV (Tables S1, S2). The specificity for each primer/probe set was confirmed using 50 ng of a pool of healthy control DNA, and those with the false positive rate of 1E-4 or higher were excluded. Subsequently, quantitative ability and sensitivity was confirmed using serial dilutions of tumor DNA or synthetic double-strand DNA (gBlocks® Gene Fragments; IDT, Coralville, IA) into a pool of five healthy control DNA. The serial dilutions ranged from 1E-2 to 1E-5 and were tested in triplicate. The primer/probe set with sensitivity 1E-4 or lower was adopted. MRD positivity was defined as a result that was both more than the sensitivity of each primer/probe set and consistent with at least one replicate. MRD negativity was defined as not positive.

### Analysis of concordances and discordances between qPCR and ddPCR-MRD

Discordances were classified according to the following criteria as previously described [39]. Discordances between positive and negative results were classified as qualitative discordances. It was also defined as major qualitative discordance when the positive result was ≥ 1E-4, and as minor qualitative discordance when the positive result was < 1E-4. A quantitative discordance was defined when both results were positive or negative, but with quantitative discrepancy > 1 log.

The correlation of MRD between qPCR and ddPCR was determined using Spearman’s rank correlation coefficient. A *P*-value < 0.05 was considered statistically significant.

## Results

### The quality of primer/probe sets of ddPCR

Of the 48 primer/probe sets of ddPCR designed for eight patients with T-ALL and four pediatric cancer patients who had their ovarian tissue cryopreserved, 22 primer/probe sets satisfied the accuracy criteria (Table S2). Twenty-two primer/probe sets failed to satisfy the criteria due to false positive rates, and four primer/probe sets were rejected due to having less sensitivity than the criteria. As a result, six out of eight patients with T-ALL and all four patients with other pediatric cancers could measure MRD using two different primer/probe sets of ddPCR targeting each SNV. In the other two patients, MRD was analyzed by one primer/probe set of ddPCR. Tumor-specific SNVs for each patient with T-ALL detected by WES are listed in Table S3.

### MRD analysis of T-ALL patients

**Fig. 1:**
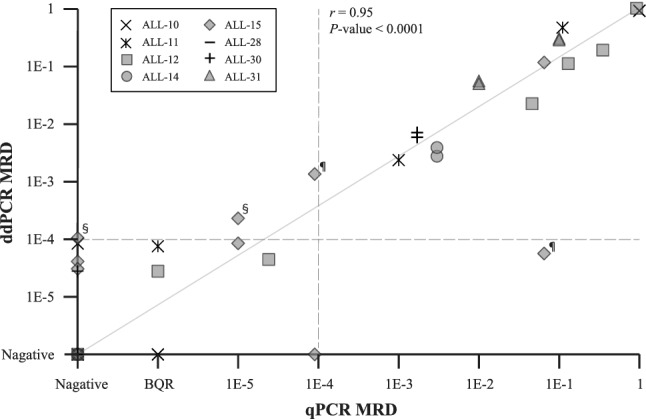
Correlation of MRD between qPCR and ddPCR. Spearman’s correlation shows a significant concordance (*r* = 0.95, *P* < 0.0001). Thirteen time points out of 26 time points were scored positive by one method or both methods, 11 of which were fully concordantly positive. The other 13 time points were scored negative by one method or both methods, including three time points below the quantitative range (BQR) of qPCR. The wavy line is on 1E-4. ¶, Major qualitative discordance; §, ddPCR-MRD negative due to less sensitivity than primer/probe set

**Fig. 2:**
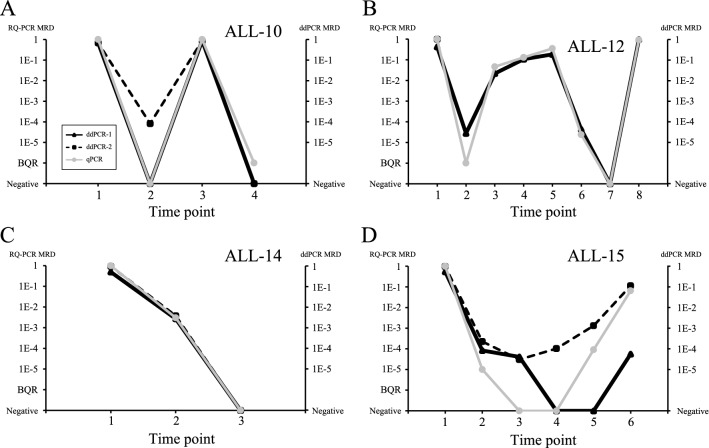
Representative results of MRD detection discordances in the follow-up samples of T-ALL patients. Comparison of MRD level evaluated by qPCR (gray lines with circle points) and ddPCR (black square or triangle). First time point indicates the onset of disease. **A, C–D**: Two different primer/probe sets for ddPCR were used in each case. **B**: One primer/probe set for ddPCR was used. **A–C**: Patients with fully concordant follow-up samples. **D**: A patient in which the results of two ddPCR MRDs deviated through the medical course. The results of second, third and fourth time point were all negative less than the primer/probe sensitivity (Table S2). ddPCR-1 was positive at the fifth time point, but PRC-MRD was negative. ddPCR-2 was negative at the sixth time point, but PCR-MRD was positive

Using these primer/probe sets, MRD evaluation was performed by both qPCR and ddPCR on 26 follow-up samples from eight patients with T-ALL. In all, we performed 26 assays using qPCR and 47 assays using ddPCR and included them in the concordance analysis (Figs. 1, 2, and S1).

The correlation coefficient of MRD between qPCR and ddPCR was 0.95 and highly significant (*P* < 0.0001) (Fig. 1). Thirty of the 41 follow-up points (73.2%) were concordant between the two methods. Major qualitative discordance was found in two of the 41 follow-up points (4.8%). Both discordances were found in the MRD analysis of one patient (ALL-15) (Figs. 1, 2). One sample of the patient at the fifth time point was negative by qPCR analysis, but one of the two primer/probe sets of ddPCR showed positive at the level of 1E-3 (Fig. 2D). Another sample of ALL-15 at the sixth time point was positive by PCR-MRD but one of the two primer/probe sets of ddPCR showed negative, and the patient suffered a relapse later on. Nine quantitative discordances of the 41 follow-up points (22%) were also found in the range below BQR of qPCR. No minor qualitative discordance was recognized.

### MRD analysis of stored ovarian tissues

We then assessed potential contamination of malignant cells in stored ovarian tissues using ddPCR-MRD.

**Fig. 3:**
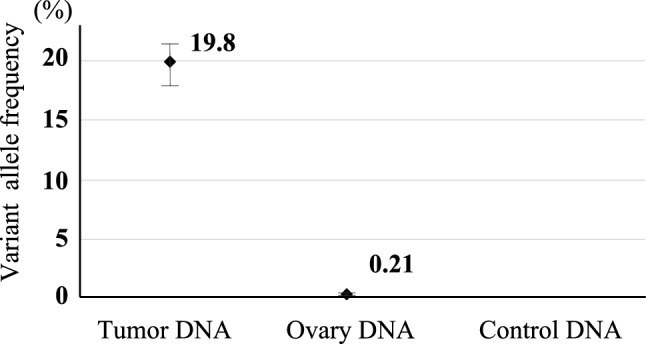
Results of ddPCR MRD positive case of ovarian tissue. The tumor-specific SNV (*WDR87* c.G8501A) was observed in 19.8% of tumor cells in UPN1 and detected in ovarian tissues at a variant allele frequency of 0.21%. Error bars represent 95% confidence intervals

Table 1 shows the clinical information of four pediatric cancer patients who underwent OTC and the results of MRD analysis of the ovarian tissues using ddPCR. The fraction of the tumor-specific SNV for UPN1 in ovarian cortex cells was 0.21%, which displayed MRD level at 1E-2 (Table 1 and Fig. 3), although MRD both in the same tissue by pathological evaluation and in the bone marrow at the same time point were negative. Reliable MRD analysis using another primer/probe set targeted another SNV for UPN1 could not be performed due to insufficient amount of tumor DNA obtained from FFPE tissue. Multiple ovarian tissues were all negative by ddPCR analysis in other cases.

## Discussion

We demonstrated that ddPCR-MRD was a highly-quantitative method to detect MRD. Considering the universal nature of this method targeting tumor-specific SNVs, the ddPCR-MRD analysis could be adopted regardless of tumor type, as shown in our analysis of stored ovarian tissues.

The results between conventional PCR-MRD targeting Ig/TCR rearrangements and ddPCR targeting tumor-specific SNVs were highly correlated, but some discordances were observed. The two major qualitative discordance suggested that clonal evolution of tumor cells led to the false negative due to disappearance of tumor-specific SNV targets (Fig. S2). In accordance with conventional PCR-MRD recommendations [37], multiple targets should be used for MRD evaluation using ddPCR in order not to miss residual minor clones or relapse by clonal evolution. Regarding this pitfall, ddPCR-MRD has an advantage because the method can increase targets for other SNVs by including passenger mutations. For ALL patient, ddPCR-MRD is useful in cases where multiple primer/probe sets targeting the Ig/TCR genes cannot be designed.

Conventional PCR-MRD using qPCR requires a large amount of diagnostic DNA because a reference standard curve is required for each measurement, limiting the number of measurements. However, ddPCR-MRD can be performed repeatedly if required, clinically utilizing its characteristic of absolute quantification of ddPCR. The sensitivity of ddPCR-MRD can go up to 10^–5^ based on previous study [16], which may greater than the sensitivity of qPCR. In addition, PCR-MRD and FCM-MRD rarely find the target for MRD detection of AML, and ddPCR-MRD might complement them, which could lead to its be applied to AML, lymphoma, and other solid tumors. Furthermore, ddPCR assay is fast, simple and easy-to-use compared to high-throughput sequencing.

Maximizing the advantage of ddPCR-MRD, we further analyzed OTC and detected contamination of malignant cells. OTC has already become an available option for fertility preservation, particularly for prepubertal cancer patients [40, 41]. Although some cancer survivors have succeeded in pregnancy and delivery after OTC and transplantation [42–48], there is a risk of malignant cell transmission [49, 50]. Histology and immunohistochemistry (IHC) and PCR-MRD have been the mainstream to detect ovarian tissue contamination by tumor cells, but histology and IHC has been suggested to be insufficient compared to PCR-MRD [50–53]. Moreover, PCR-MRD cannot be used for MRD detection in tumors lacking of a monoclonal molecular marker. In previous studies of cancer patients who underwent OTC, molecular markers for PCR were available in 28%-89% of leukemia patients and 39% of bone and soft tissue sarcoma patients [50, 54, 55]. Nguyen TYT et al. analyzed MRD in ovarian tissues from 20 patients with central nervous system tumors who underwent OTC by ddPCR targeting glial fibrillary acidic protein (GFAP) and neuron-specific enolase (NSE) [56]. Our method using ddPCR also could analyze MRD of preserved ovarian tissues in all four cases and detect MRD contamination in one case. ddPCR might contribute to safer transplantation of cryopreserved ovarian tissue.

This study has several limitations. First, due to the small sample size, clinical significance of ddPCR-MRD as a prognostic factor is not established. Further studies including a large number of cases should be performed. Second, the MRD of ovarian tissue was not strictly an evaluation of the whole tissue to be transplanted. Clinical significance of positivity and negativity of MRD in ovarian tissues should be also confirmed by further studies.

In conclusion, we demonstrated a sensitive MRD assay using ddPCR targeting tumor-specific SNVs. Considering the advantage of ddPCR-MRD in universality, the method has the potential to be applied as a complement for not only ALL, but also other malignant diseases regardless of tumor-specific markers, including tumor contamination screening of cryopreserved ovarian tissues.

## Supplementary Information

Below is the link to the electronic supplementary material.

Supplementary file1 (PDF 324 KB)

## Data Availability

The data that support the findings of this study are available on request.

## References

[1] Coustan-Smith E, Behm FG, Sanchez J, Boyett JM, Hancock ML, Raimondi SC (1998). Immunological detection of minimal residual disease in children with acute lymphoblastic leukaemia. Lancet.

[2] Pui CH, Pei D, Coustan-Smith E, Jeha S, Cheng C, Bowman WP (2015). Clinical utility of sequential minimal residual disease measurements in the context of risk-based therapy in childhood acute lymphoblastic leukaemia: a prospective study. Lancet Oncol.

[3] van Dongen JJ, Seriu T, Panzer-Grümayer ER, Biondi A, Pongers-Willemse MJ, Corral L (1998). Prognostic value of minimal residual disease in acute lymphoblastic leukaemia in childhood. Lancet.

[4] Grimwade D, Jovanovic JV, Hills RK, Nugent EA, Patel Y, Flora R (2009). Prospective minimal residual disease monitoring to predict relapse of acute promyelocytic leukemia and to direct pre-emptive arsenic trioxide therapy. J Clin Oncol: Off J Am Soc Clin Oncol.

[5] Platzbecker U, Middeke JM, Sockel K, Herbst R, Wolf D, Baldus CD (2018). Measurable residual disease-guided treatment with azacitidine to prevent haematological relapse in patients with myelodysplastic syndrome and acute myeloid leukaemia (RELAZA2): an open-label, multicentre, phase 2 trial. Lancet Oncol.

[6] Kurtz DM, Green MR, Bratman SV, Scherer F, Liu CL, Kunder CA (2015). Noninvasive monitoring of diffuse large B-cell lymphoma by immunoglobulin high-throughput sequencing. Blood.

[7] Roschewski M, Dunleavy K, Pittaluga S, Moorhead M, Pepin F, Kong K (2015). Circulating tumour DNA and CT monitoring in patients with untreated diffuse large B-cell lymphoma: a correlative biomarker study. Lancet Oncol.

[8] Scherer F, Kurtz DM, Newman AM, Stehr H, Craig AF, Esfahani MS (2016). Distinct biological subtypes and patterns of genome evolution in lymphoma revealed by circulating tumor DNA. Sci Transl Med..

[9] Marachelian A, Villablanca JG, Liu CW, Liu B, Goodarzian F, Lai HA (2017). Expression of five neuroblastoma genes in bone marrow or blood of patients with relapsed/refractory neuroblastoma provides a new biomarker for disease and prognosis. Clin Cancer Res: Off J Am Assoc Cancer Res.

[10] Moss TJ, Sanders DG (1990). Detection of neuroblastoma cells in blood. J Clin Oncol: Off J Am Soc Clin Oncol.

[11] Brüggemann M, Schrauder A, Raff T, Pfeifer H, Dworzak M, Ottmann OG (2010). Standardized MRD quantification in European ALL trials: proceedings of the second international symposium on MRD assessment in Kiel, Germany, 18–20 September 2008. Leukemia.

[12] van Dongen JJ, Langerak AW, Brüggemann M, Evans PA, Hummel M, Lavender FL (2003). Design and standardization of PCR primers and protocols for detection of clonal immunoglobulin and T-cell receptor gene recombinations in suspect lymphoproliferations: report of the BIOMED-2 concerted action BMH4-CT98-3936. Leukemia.

[13] Chen X, Wood BL (2017). How do we measure MRD in all and how should measurements affect decisions. Re: treatment and prognosis?. Best Pract Res Clin Haematol..

[14] Denys B, van der Sluijs-Gelling AJ, Homburg C, van der Schoot CE, de Haas V, Philippé J (2013). Improved flow cytometric detection of minimal residual disease in childhood acute lymphoblastic leukemia. Leukemia.

[15] Boeckx N, Willemse MJ, Szczepanski T, van der Velden VH, Langerak AW, Vandekerckhove P (2002). Fusion gene transcripts and Ig/TCR gene rearrangements are complementary but infrequent targets for PCR-based detection of minimal residual disease in acute myeloid leukemia. Leukemia.

[16] Della Starza I, Chiaretti S, De Propris MS, Elia L, Cavalli M, De Novi LA (2019). Minimal residual disease in acute lymphoblastic leukemia: technical and clinical advances. Front Oncol.

[17] Della Starza I, Nunes V, Lovisa F, Silvestri D, Cavalli M, Garofalo A (2018). Digital-droplet PCR, an accurate method for IG/TR PCR-MRD stratification in childhood acute lymphoblastic leukemia. Blood..

[18] Kotrova M, Muzikova K, Mejstrikova E, Novakova M, Bakardjieva-Mihaylova V, Fiser K (2015). The predictive strength of next-generation sequencing MRD detection for relapse compared with current methods in childhood all. Blood.

[19] van Dongen JJ, van der Velden VH, Brüggemann M, Orfao A (2015). Minimal residual disease diagnostics in acute lymphoblastic leukemia: need for sensitive, fast, and standardized technologies. Blood.

[20] Della Starza I, Nunes V, Cavalli M, De Novi LA, Ilari C, Apicella V (2016). Comparative analysis between RQ-PCR and digital-droplet-PCR of immunoglobulin/T-cell receptor gene rearrangements to monitor minimal residual disease in acute lymphoblastic leukaemia. Br J Haematol.

[21] Logan AC, Vashi N, Faham M, Carlton V, Kong K, Buño I (2014). Immunoglobulin and T cell receptor gene high-throughput sequencing quantifies minimal residual disease in acute lymphoblastic leukemia and predicts post-transplantation relapse and survival. Biol Blood Marrow Transpl: J Am Soc Blood Marrow Transpl.

[22] Bartram J, Wright G, Adams S, Archer P, Brooks T, Edwards D (2022). High-throughput sequencing of peripheral blood for minimal residual disease monitoring in childhood precursor B-cell acute lymphoblastic leukemia: a prospective feasibility study. Pediatr Blood Cancer..

[23] Della Starza I, De Novi LA, Santoro A, Salemi D, Tam W, Cavalli M (2019). Digital droplet PCR and next-generation sequencing refine minimal residual disease monitoring in acute lymphoblastic leukemia. Leuk Lymphoma.

[24] Grassi S, Guerrini F, Ciabatti E, Puccetti R, Salehzadeh S, Metelli MR (2020). Digital droplet PCR is a specific and sensitive tool for detecting IDH2 mutations in acute myeloid leukemia patients. Cancers..

[25] Ansuinelli M, Della Starza I, Lauretti A, Elia L, Siravo V, Messina M (2021). Applicability of droplet digital polymerase chain reaction for minimal residual disease monitoring in Philadelphia-positive acute lymphoblastic leukaemia. Hematol Oncol.

[26] Coccaro N, Anelli L, Zagaria A, Casieri P, Tota G, Orsini P (2018). Droplet digital PCR is a robust tool for monitoring minimal residual disease in adult philadelphia-positive acute lymphoblastic leukemia. J Mol Diagn.

[27] Saliba J, Evensen NA, Meyer JA, Newman D, Nersting J, Narang S (2020). Feasibility of monitoring peripheral blood to detect emerging clones in children with acute lymphoblastic leukemia(†). Pediatr Blood Cancer..

[28] Kawamura K, Cheng Y, Suzuki N, Deguchi M, Sato Y, Takae S (2013). Hippo signaling disruption and Akt stimulation of ovarian follicles for infertility treatment. Proc Natl Acad Sci USA.

[29] Suzuki N, Yoshioka N, Takae S, Sugishita Y, Tamura M, Hashimoto S (2015). Successful fertility preservation following ovarian tissue vitrification in patients with primary ovarian insufficiency. Hum Reprod (Oxford, England).

[30] Fukawatase Y, Toyoda M, Okamura K, Nakamura K, Nakabayashi K, Takada S (2014). Ataxia telangiectasia derived iPS cells show preserved x-ray sensitivity and decreased chromosomal instability. Sci Rep.

[31] Kumar P, Henikoff S, Ng PC (2009). Predicting the effects of coding non-synonymous variants on protein function using the SIFT algorithm. Nat Protoc.

[32] Adzhubei I, Jordan DM, Sunyaev SR (2013). Predicting functional effect of human missense mutations using PolyPhen-2. Curr Protoc Hum Genet..

[33] Kircher M, Witten DM, Jain P, O'Roak BJ, Cooper GM, Shendure J (2014). A general framework for estimating the relative pathogenicity of human genetic variants. Nat Genet.

[34] Eckert C, Henze G, Seeger K, Hagedorn N, Mann G, Panzer-Grümayer R (2013). Use of allogeneic hematopoietic stem-cell transplantation based on minimal residual disease response improves outcomes for children with relapsed acute lymphoblastic leukemia in the intermediate-risk group. J Clin Oncol: Off J Am Soc Clin Oncol.

[35] Szczepański T, Flohr T, van der Velden VH, Bartram CR, van Dongen JJ (2002). Molecular monitoring of residual disease using antigen receptor genes in childhood acute lymphoblastic leukaemia. Best Pract Res Clin Haematol.

[36] Flohr T, Schrauder A, Cazzaniga G, Panzer-Grümayer R, van der Velden V, Fischer S (2008). Minimal residual disease-directed risk stratification using real-time quantitative PCR analysis of immunoglobulin and T-cell receptor gene rearrangements in the international multicenter trial AIEOP-BFM ALL 2000 for childhood acute lymphoblastic leukemia. Leukemia.

[37] van der Velden VH, Cazzaniga G, Schrauder A, Hancock J, Bader P, Panzer-Grumayer ER (2007). Analysis of minimal residual disease by Ig/TCR gene rearrangements: guidelines for interpretation of real-time quantitative PCR data. Leukemia.

[38] Drandi D, Alcantara M, Benmaad I, Söhlbrandt A, Lhermitte L, Zaccaria G (2020). Droplet digital PCR quantification of mantle cell lymphoma follow-up samples from four prospective trials of the European MCL network. HemaSphere..

[39] Ladetto M, Brüggemann M, Monitillo L, Ferrero S, Pepin F, Drandi D (2014). Next-generation sequencing and real-time quantitative PCR for minimal residual disease detection in B-cell disorders. Leukemia.

[40] Lee SJ, Schover LR, Partridge AH, Patrizio P, Wallace WH, Hagerty K (2006). American Society of Clinical Oncology recommendations on fertility preservation in cancer patients. J Clin Oncol: Off J Am Soc Clin Oncol.

[41] Balduzzi A, Dalle JH, Jahnukainen K, von Wolff M, Lucchini G, Ifversen M (2017). Fertility preservation issues in pediatric hematopoietic stem cell transplantation: practical approaches from the consensus of the pediatric diseases working party of the EBMT and the international BFM study group. Bone Marrow Transpl.

[42] Donnez J, Dolmans MM, Demylle D, Jadoul P, Pirard C, Squifflet J (2004). Livebirth after orthotopic transplantation of cryopreserved ovarian tissue. Lancet.

[43] Jensen AK, Kristensen SG, Macklon KT, Jeppesen JV, Fedder J, Ernst E (2015). Outcomes of transplantations of cryopreserved ovarian tissue to 41 women in Denmark. Hum Reprod (Oxford, England).

[44] Meirow D, Ra'anani H, Shapira M, Brenghausen M, Derech Chaim S, Aviel-Ronen S (2016). Transplantations of frozen-thawed ovarian tissue demonstrate high reproductive performance and the need to revise restrictive criteria. Fertil Steril.

[45] Donnez J, Dolmans MM (2017). Fertility preservation in women. N Engl J Med.

[46] Shapira M, Raanani H, Barshack I, Amariglio N, Derech-Haim S, Marciano MN (2018). First delivery in a leukemia survivor after transplantation of cryopreserved ovarian tissue, evaluated for leukemia cells contamination. Fertil Steril.

[47] Silber SJ, DeRosa M, Goldsmith S, Fan Y, Castleman L, Melnick J (2018). Cryopreservation and transplantation of ovarian tissue: results from one center in the USA. J Assist Reprod Genet.

[48] Hossay C, Donnez J, Dolmans MM (2020). Whole ovary cryopreservation and transplantation: a systematic review of challenges and research developments in animal experiments and humans. J Clin Med..

[49] Bastings L, Beerendonk CC, Westphal JR, Massuger LF, Kaal SE, van Leeuwen FE (2013). Autotransplantation of cryopreserved ovarian tissue in cancer survivors and the risk of reintroducing malignancy: a systematic review. Hum Reprod Update.

[50] Dolmans MM, Marinescu C, Saussoy P, Van Langendonckt A, Amorim C, Donnez J (2010). Reimplantation of cryopreserved ovarian tissue from patients with acute lymphoblastic leukemia is potentially unsafe. Blood.

[51] Meirow D, Hardan I, Dor J, Fridman E, Elizur S, Ra'anani H (2008). Searching for evidence of disease and malignant cell contamination in ovarian tissue stored from hematologic cancer patients. Hum Reprod (Oxford, England).

[52] Rosendahl M, Greve T, Andersen CY (2013). The safety of transplanting cryopreserved ovarian tissue in cancer patients: a review of the literature. J Assist Reprod Genet.

[53] Soares M, Sahrari K, Amorim CA, Saussoy P, Donnez J, Dolmans MM (2015). Evaluation of a human ovarian follicle isolation technique to obtain disease-free follicle suspensions before safely grafting to cancer patients. Fertil Steril.

[54] Dolmans MM, Iwahara Y, Donnez J, Soares M, Vaerman JL, Amorim CA (2016). Evaluation of minimal disseminated disease in cryopreserved ovarian tissue from bone and soft tissue sarcoma patients. Hum Reprod (Oxford, England).

[55] Soares M, Saussoy P, Maskens M, Reul H, Amorim CA, Donnez J (2017). Eliminating malignant cells from cryopreserved ovarian tissue is possible in leukaemia patients. Br J Haematol.

[56] Nguyen TYT, Cacciottola L, Camboni A, Ravau J, De Vos M, Demeestere I (2021). Ovarian tissue cryopreservation and transplantation in patients with central nervous system tumours. Hum Reprod (Oxford, England).

