# Supplementary material for: Minimal residual disease detection by mutation-specific droplet digital PCR for leukemia/lymphoma
Source: Int J Hematol. 2023 Mar 3;117(6):910–8. doi: 10.1007/s12185-023-03566-2 (PMC10225364; doi:10.1007/s12185-023-03566-2)
Supplement: Supplementary file 1 — Supplementary file1 (PDF 324 KB) [file 12185_2023_3566_MOESM1_ESM.pdf]

Supporting Information

Table S1. Single nucleotide variant targeted for analysis of minimal residual disease

Case	Gene	Position	Annotation	Allele frequency
UPN1	*PLXND1*	Chr3: 129324713	c.T770G: p.L257R: NM_015103	0.25
UPN1	*WDR87*	Chr19: 38375693	c.G8501A: p.R2834H: NM_031951	0.22
UPN2	*KCTD7*	Chr7: 66103989	c.C640T: p.R214W: NM_001167961	0.48
UPN2	*FLT3*	Chr13: 28592642	c.G2503T: p.D835Y: NM_004119	0.49
UPN3	*UGGT1*	Chr2: 128886743	c.C1367T: p.P456L: NM_020120	0.24
UPN3	*ACO1*	Chr9: 32450036	c.A2597T: p.D866V: NM_002197	0.28
UPN4	*PCLO*	Chr7 :82585006	c.C5263T: p.Q1755X: NM_014510	0.31
UPN4	*RMI1*	Chr9: 86616417	c.T516C: p.N172N: NM_024945	0.13
ALL-10	*TRAF1*	Chr9: 123675707	c.G238T: p.A80S: NM_001190947	0.29
ALL-10	*PLEKHG4B*	Chr5: 143595	c.G720T: p.L240L: NM_052909	0.41
ALL-11	*MTO1*	Chr6: 74171711	c.G134C: p.G45A: NM_001123226	0.39
ALL-12	*KDM6A*	ChrX: 44942752	c.G3095C: p.R1032P: NM_001291418	0.37
ALL-14	*ATP10D*	Chr4: 47559954	c.G2098C: p.G700R: NM_020453	0.37
ALL-14	*CES-1*	Chr16: 55846832	c.G1066C: p.G356R: NM_001025194	0.39
ALL-15	*GPR97*	Chr16: 57717942	c.T980C: p.V327A: NM_170776	0.5
ALL-15	*STAT5A*	Chr17: 40457661	c.G1324C: p.G442R: NM_001288719	0.27
ALL-28	*ARIDIA*	Chr1: 27023581	c.C687G: p.Y229X: NM_006015	0.51
ALL-28	*NOTCH1*	Chr9: 139390791	c.C7400A: p.S2467X: NM_017617	0.53
ALL-30	*ADAM22*	Chr7: 2387792403	c.A1984C: p.M662L: NM_004194	0.20
ALL-30	*TTC28*	Chr22: 28379198	c.A6457G: p.S2153G: NM_001145418	0.18
ALL-31	*FUOM*	Chr10: 135169275	c.G296A: p.R99Q: NM_001301827	0.40
ALL-31	*DNM2*	Chr19: 10906762	c.G1222C: p.A408P: NM_001005360	0.43

Table S2: Primer and probe sequences to analyze minimal residual disease for digital droplet PCR

Case	Gene	Primer sequence (5'>3')	Probe sequence (5'>3')	Annealing temperature (ºC)	Sensitivity
UPN1	*PLXND1*	Forward: CCTGGCCAAGCTCT<br>Reverse: GATCTTGAGGATGTTGTCGT	Wild type: /5HEX/CCTTCGACC/Zen/TCAACCC/3IABkFQ/<br>Mutant: /56-FAM/CCTTCGACC/Zen/GCAACC/3IABkFQ/	58	0.0001
UPN1	*WDR87*	Forward: GGTACCCCTCCCATG<br>Reverse: CCCATACCGGGCAAT	Wild type: /5HEX/CTGTGTGCG/Zen/CACCATC/3IABkFQ/<br>Mutant: /56-FAM/TGTGTGCAC/Zen/ACCATCCT/3IABkFQ/	56	0.00027
UPN2	*KCTD7*	Forward: CCGCTCCTCAACTCC<br>Reverse: GGTGCTCAAAAAGCTGC	Wild type: /5HEX/CTCACTCCG/Zen/CTCAAATCG/3IABkFQ/<br>Mutant: /56-FAM/CTCTCACTC/Zen/CACTCAAATCG/3IABkFQ/	58	0.00078
UPN2	*FLT3*	Forward: TGGTGAAGATATGTGACTTTGG<br>Reverse: ATTGCCCCTGACAACATAG	Wild type: /5HEX/TGGCTCG+A+G+ATATCA/3IABkFQ/<br>Mutant: /56-FAM/TGGC+TCG+A+T+ATA+TCAT/3IABkFQ/	58	0.00093
UPN3	*UGGT1*	Forward: TCTGAGGCAGACTATGC<br>Reverse: TCCTTTGATCACCCTAACA	Wild type: /5HEX/ATAGCAGGA/Zen/CTCCGGA/3IABkFQ/<br>Mutant: /56-FAM/ATAGCAAGA/Zen/CTCCGGATG/3IABkFQ/	58	0.00061
UPN3	*ACO1*	Forward: CAAGACCTTCCAGGCT<br>Reverse: CCGTTGAGGAAATAAGTGAG	Wild type: /5HEX/CCACATCAG/Zen/TGTCAAACCT/3IABkFQ/<br>Mutant: /56-FAM/CCACATCAG/Zen/TGACAAACCT/3IABkFQ/	56	0.00032
UPN4	*PCLO*	Forward: GTAGCCCGAGTCACAA<br>Reverse: AAGAGGGCCATGTGG	Wild type: /5HEX/TGCGT+T+GC+TGTTT/3IABkFQ/<br>Mutant: /56-FAM/TGCGT+T+A+CTGTTTGC/3IABkFQ/	54	0.0001
UPN4	*RMI1*	Forward: CTTCCTCCAGGTACAAAAAT<br>Reverse: ACGTTTTCTGGTTTCAATAAG	Wild type: /5HEX/TGGAA+A+T+ATAT+CTT+TCCG/3IABkFQ/<br>Mutant: /56-FAM/TGGAA+A+C+ATAT+CTT+TCC/3IABkFQ/	53	0.00054
ALL-10	*TRAF1*	Forward: AAGCTGCGTGTGTTTG<br>Reverse: GGATAGAGGTGGCCAG	Wild type: /5HEX/AACATTGTT/ZEN/GCTGTCCTCA/3IABkFQ/<br>Mutant: /56-FAM/ACATTGTTT/ZEN/CTGTCCTCAACA/3IABkFQ/	57	0.00013

ALL-10	*PLEKHG4B*	Forward: TCGTCCTGTGCTGAG<br>Reverse: CACCTGGGGATGCTAT	Wild type: /5HEX/GCCTGCTGC/ZEN/TGTACT/3IABkFQ/<br>Mutant: /56-FAM/CGCCTGCTT/ZEN/CTGTACT/3IABkFQ/	52	0.00028
ALL-11	*MTO1*	Forward: GACTCCGCACTTCGAC<br>Reverse: GCAGAGTCCGAGAGC	Wild type: /5HEX/CCGGCATGT/ZEN/CCTCCA/3IABkFQ/<br>Mutant: /56-FAM/CGGCATGTG/ZEN/CTCCAC/3IABkFQ/	55	0.00072
ALL-12	*KDM6A*	Forward: ACATGAGCTGACTAAACTTC<br>Reverse: GACCAACATGGCTTAGAAG	Wild type: /5HEX/TGTGCGTGT/ZEN/CGTATCAGC/3IABkFQ/<br>Mutant: /56-FAM/TGTGCCTGT/ZEN/CGTATCAG/3IABkFQ/	57	0.00077
ALL-14	*CES-1*	Forward: CCCCTACATGGTCGGA<br>Reverse: CATGTGCCTTCTCACCAT	Wild type: /5HEX/CCAGCCAAA/ZEN/CTCCTGC/3IABkFQ/<br>Mutant: /56-FAM/CCAGCGAAA/ZEN/CTCCTGC/3IABkFQ/	57	0.0002
ALL-14	*ATP10D*	Forward: GCTTGCTGCACAGAAAC<br>Reverse: GACTCTGCCTTGCCATT	Wild type: /5HEX/CTGCATCAC/ZEN/CGTGTTGTT/3IABkFQ/<br>Mutant: /56-FAM/CTGCATCAC/ZEN/GGTGTTGT/3IABkFQ/	52	0.00017
ALL-15	*STAT5A*	Forward: GACTCTGTCCCTACCTGT<br>Reverse: ACAGTAGCCGTGGCA	Wild type: /5HEX/CTGCCGTGG/ZEN/ACGATGA/3IABkFQ/<br>Mutant: /56-FAM/CTGCGGTGG/ZEN/ACGATGA/3IABkFQ/	55	0.00053
ALL-15	*GPR97*	Forward: CCTCCTGAATCTGGCC<br>Reverse: GGCAGCATCAGACCC	Wild type: /5HEX/ACTCCCCAC/ZEN/ATTGACCA/3IABkFQ/<br>Mutant: /56-FAM/TCCCCGCAT/ZEN/TGACCA/3IABkFQ/	55	0.00032
ALL-28	*ARIDIA*	Forward: AGTACAACTCCTACTACCCC<br>Reverse: GAGTGCCACCTCTCG	Wild type: /5HEX/CCTACGCGC/Zen/TGAGCT/3IABkFQ/<br>Mutant: /56-FAM/CCTAGGCGC/Zen/TGAGCT/3IABkFQ/	58	0.00031
ALL-28	*NOTCH1*	Forward: TCTGCCCCAGGAGAG<br>Reverse: GTCAGGAACTGGGCTG	Wild type: /5HEX/ATGGCAGCG/Zen/ACGTGG/3IABkFQ/<br>Mutant: /56-FAM/TGGCAGCTA/Zen/CGTGGG/3IABkFQ/	58	0.00034
ALL-30	*ADAM22*	Forward: ATGGGACACCTTGTG<br>Reverse: CACAGGAAGACACCT	Wild type: /5HEX/AGCACATCA/Zen/TTTGGGG/3IABkFQ<br>Mutant: /56-FAM/AGCACAGCA/Zen/TTTGGG/3IABkFQ/	55	0.00012
ALL-30	*TTC28*	Forward: GGACAGTACCGTGAAAT<br>Reverse: GGGCTAACTCTTGTGG	Wild type: /5HEX/CAAGAAGAA/Zen/AGCAACCCA/3IABkFQ/<br>Mutant: /56-FAM/AGAAGAAGG/Zen/CAACCCA/3IABkFQ/	55	0.00018

ALL-31	*FUOM*	Forward: CCTGGCAAAGATAGAGAGG<br>Reverse: CGTTGCCACAACAGC	Wild type: /5HEX/ATGAA+C+G+GGCTAAG/3IABkFQ/<br>Mutant: /56-FAM/TATG+AA+C+A+GGC+TAAGA/3IABkFQ/	55	0.00025
ALL-31	*DNM2*	Forward: ACCGGGCTTTTCACC<br>Reverse: TCAGCTTGACGACCTG	Wild type: /5HEX/CTCGAATGC/Zen/CAAGTCCGG/3IABkFQ/<br>Mutant: /56-FAM/TCG+AAT+G+G+CAAGTC/3IABkFQ/	53	0.00079

Table S3. Tumor specific single nucleotide variants in T-ALL patients detected by whole exome sequencing

Case	Gene	Position	Annotation	Allele frequency
ALL-10	*KCNV2*	chr9: 2717871	NM_133497:c.C132T:p.H44H	0.28
ALL-10	*TRAF1*	chr9: 123675707	NM_001190947:c.G238T:p.A80S	0.29
ALL-10	*PKHD1L1*	chr8: 110510958	NM_177531:c.A10786G:p.I3596V	0.32
ALL-10	*PLEKHG4B*	chr5: 143595	NM_052909:c.G720T:p.L240L	0.41
ALL-10	*ENPP1*	chr6: 132201162	NM_006208:c.C2088T:p.T696T	0.38
ALL-10	*ACSS3*	chr12: 81624909	NM_024560:c.A1588C:p.K530Q	0.39
ALL-10	*TAPBPL*	chr12: 6571306	NM_018009:c.G1398C:p.Q466H	0.34
ALL-10	*THNSL1*	chr10: 25313553	NM_024838:c.G1401A:p.S467S	0.37
ALL-10	*TMEM27*	chrX: 15663066	NM_020665:c.C290A:p.P97H	0.22
ALL-10	*NRAS*	chr1: 115258745	NM_002524:c.G37C:p.G13R	0.23
ALL-10	*SCAF1*	chr19: 50161603	NM_021228:c.G3886A:p.E1296K	0.36
ALL-10	*CNN3*	chr1: 95367275	NM_001286055:c.C313T:p.R105C	0.35
ALL-10	*C1orf116*	chr1: 207195539	NM_001083924:c.C832T:p.R278C	0.34
ALL-10	*VWA5B1*	chr1: 20656717	NM_001039500:c.C1299T:p.A433A	0.27
ALL-10	*MUC17*	chr7: 100682068	NM_001040105:c.G7371A:p.P2457P	0.31
ALL-10	*RAG1*	chr11: 36595452	NM_000448:c.G598T:p.V200L	0.27
ALL-10	*RIN2*	chr20: 19972907	NM_018993:c.A2164C:p.M722L	0.01
ALL-10	*NMI*	chr2: 152132347	NM_004688:c.T372G:p.H124Q	0.10
ALL-11	*USP31*	chr16: 23160473	NM_020718:c.G119T:p.G40V	0.44
ALL-11	*ABCA5*	chr17: 67302906	NM_018672:c.G748A:p.E250K	0.70
ALL-11	*NTM*	chr11: 132177583	NM_001144058:c.C527T:p.A176V	0.38
ALL-11	*ASB18*	chr2: 237172866	NM_212556:c.G123A:p.T41T	0.37
ALL-11	*TTYH1*	chr19: 54946839	NM_001005367:c.C1243T:p.R415X	0.58
ALL-11	*NOTCH3*	chr19: 15271843	NM_000435:c.G6596A:p.G2199E	0.65
ALL-11	*YTHDC1*	chr4: 69188491	NM_133370:c.G1523A:p.R508Q	0.45
ALL-11	*MTO1*	chr6: 74171711	NM_001123226:c.G134C:p.G45A	0.39
ALL-11	*SLC35G1*	chr10: 95661222	NM_001134658:c.G1073A:p.R358H	0.44
ALL-11	*SLC12A1*	chr15: 48559758	NM_000338:c.G2155A:p.G719R	0.45
ALL-11	*PPP2R3A*	chr3: 135745756	NM_181897:c.C215T:p.P72L	0.34
ALL-11	*MCF2L*	chr13: 113729318	NM_001112732:c.G1123A:p.V375M	0.33
ALL-11	*ANK3*	chr10: 61846555	NM_001149:c.C1030T:p.R344W	0.23
ALL-11	*KALRN*	chr3: 124175525	NM_001024660:c.C3798T:p.D1266D	0.36
ALL-11	*LUZP1*	chr1: 23418182	NM_001142546:c.C2573T:p.A858V	0.25
ALL-12	*RBM46*	chr4: 155719362	NM_001277171:c.G551A:p.R184H	0.46
ALL-12	*COL5A2*	chr2: 189917479	NM_000393:c.C2711T:p.P904L	0.42
ALL-12	*ADD2*	chr2: 70933506	NM_001185055:c.C83T:p.P28L	0.45
ALL-12	*DOCK6*	chr19: 11312669	NM_020812:c.G5584A:p.A1862T	0.28
ALL-12	*KDM6A*	chrX: 44942752	NM_001291418:c.G3095C:p.R1032P	0.37
ALL-12	*SZT2*	chr1: 43905631	NM_015284:c.C6951A:p.L2317L	0.41

ALL-12	*PREPL*	chr2: 44559618	NM_001042385:c.C1147T:p.R383C	0.39
ALL-12	*SCRIB*	chr8: 144874945	NM_015356:c.C4110T:p.A1370A	0.37
ALL-12	*GRHL3*	chr1: 24658015	NM_021180:c.G132A:p.P44P	0.43
ALL-12	*TFR2*	chr7: 100225433	NM_001206855:c.C1103A:p.P368H	0.10
ALL-12	*USP2*	chr11: 119243423	NM_004205:c.C768T:p.D256D	0.12
ALL-12	*TRPC7*	chr5: 135610517	NM_001167577:c.T789C:p.A263A	0.13
ALL-12	*BTN1A1*	chr6: 26509240	NM_001732:c.C1419T:p.C473C	0.14
ALL-12	*SLX4*	chr16: 3656604	NM_032444:c.C631T:p.Q211X	0.09
ALL-12	*USP38*	chr4: 144106782	NM_001290325:c.T179G:p.V60G	0.22
ALL-12	*C10orf113*	chr10: 21435342	NM_001010896:c.T96A:p.S32R	0.05
ALL-12	*HSPA2*	chr14: 65008776	NM_021979:c.G1209A:p.S403S	0.00
ALL-12	*ZEB2*	chr2: 145147449	NM_001171653:c.C3142A:p.Q1048K	0.01
ALL-12	*OR6B1*	chr7: 143701933	NM_001005281:c.T844C:p.S282P	0.02
ALL-12	*PDE11A*	chr2: 178936689	NM_016953:c.G476A:p.R159Q	0.00
ALL-14	*ZNF516*	chr18: 74153561	NM_014643:c.C1450T:p.P484S	0.40
ALL-14	*CES1*	chr16: 55846832	NM_001025194:c.G1066C:p.G356R	0.39
ALL-14	*CORO2B*	chr15: 69007571	NM_001190456:c.G873A:p.R291R	0.39
ALL-14	*PI4KA*	chr22: 21105663	NM_058004:c.A3071G:p.D1024G	0.48
ALL-14	*SIGLEC11*	chr19: 50461951	NM_001135163:c.G1312A:p.A438T	0.28
ALL-14	*SPAG17*	chr1: 118530787	NM_206996:c.T5562A:p.A1854A	0.50
ALL-14	*ATP10D*	chr4: 47559954	NM_020453:c.G2098C:p.G700R	0.37
ALL-14	*CARD9*	chr9: 139261667	NM_052814:c.1311+1G>T	0.36
ALL-14	*GAS6*	chr13: 114538523	NM_000820:c.G675A:p.E225E	0.43
ALL-14	*PPP1R9A*	chr7: 94915612	NM_001166163:c.A2852G:p.Q951R	0.33
ALL-14	*KLHL21*	chr1: 6653431	NM_014851:c.G1788A:p.L596L	0.31
ALL-14	*TTN*	chr2: 179454748	NM_003319:c.C34509A:p.D11503E	0.33
ALL-14	*SPATA17*	chr1: 217955533	NM_138796:c.C741T:p.I247I	0.12
ALL-14	*ZNF284*	chr19: 44590834	NM_001037813:c.C1203T:p.C401C	0.11
ALL-14	*ARFGEF1*	chr8: 68179441	NM_006421:c.A1697T:p.Y566F	0.17
ALL-14	*PYY*	chr17: 42030832	NM_004160:c.C20T:p.P7L	0.13
ALL-14	*FRRS1*	chr1: 100183024	NM_001013660:c.G1178A:p.R393Q	0.07
ALL-14	*PABPC3*	chr13: 25671214	NM_030979:c.T878G:p.V293G	0.20
ALL-14	*PPL*	chr16: 4935256	NM_002705:c.A3400C:p.T1134P	0.20
ALL-14	*ETV6*	chr12: 12037406	NM_001987:c.1037_1039del:p.346_347del	0.49
ALL-14	*IFT140*	chr16: 1574650	NM_014714:c.A3044C:p.H1015P	0.45
ALL-14	*ASTN1*	chr1: 176992701	NM_001286164:c.G1277A:p.R426H	0.33
ALL-14	*HNRNPCL1*	chr1: 12907316	NM_001146181:c.A827G:p.E276G	0.30
ALL-14	*MTM1*	chrX: 149809877	NM_000252:c.C664T:p.R222X	0.28
ALL-14	*MUC17*	chr7: 100679388	NM_001040105:c.A4691C:p.Q1564P	0.22
ALL-15	*GPR97*	chr16: 57717942	NM_170776:c.T980C:p.V327A	0.50
ALL-15	*MGAT4B*	chr5: 179225241	NM_054013:c.A1592G:p.D531G	0.43
ALL-15	*NAP1L3*	chrX: 92927913	NM_004538:c.G391A:p.D131N	0.37

ALL-15	*STAT5A*	chr17: 40457661	NM_001288719:c.G1324C:p.G442R	0.27
ALL-15	*ADAMTS3*	chr4: 73181662	NM_014243:c.G1512A:p.Q504Q	0.22
ALL-28	*GPRIN2*	chr10: 46999857	NM_014696:c.A977T:p.E326V	0.18
ALL-28	*SLC22A17*	chr14: 23818582	NM_020372:c.G425T:p.G142V	0.39
ALL-28	*MYH7*	chr14: 23885011	NM_000257:c.C4984T:p.R1662C	0.39
ALL-28	*ZFP36L2*	chr2: 43451724	NM_006887:c.1218dupC:p.A407fs	0.39
ALL-28	*PHIP*	chr6: 79671428	NM_017934:c.G3635T:p.R1212I	0.40
ALL-28	*VPS4A*	chr16: 69350182	NM_013245:c.A188G:p.Y63C	0.40
ALL-28	*DPP3*	chr11: 66260241	NM_001256670:c.C953T:p.A318V	0.40
ALL-28	*DNHD1*	chr11: 6578015	NM_144666:c.C7490A:p.T2497K	0.41
ALL-28	*TAF1*	chrX: 70618501	NM_001286074:c.C3760T:p.R1254W	0.41
ALL-28	*FAT3*	chr11: 92257934	NM_001008781:c.G3427A:p.D1143N	0.42
ALL-28	*DCHS2*	chr4: 155237038	NM_017639:c.C3757A:p.Q1253K	0.44
ALL-28	*TTN*	chr2: 179398539	NM_003319:c.C75608T:p.T25203I	0.45
ALL-28	*PIAS1*	chr15: 68378809	NM_016166:c.C190T:p.R64W	0.45
ALL-28	*ENTHD2*	chr17: 79205795	NM_144679:c.G553A:p.G185R	0.48
ALL-28	*POLR2E*	chr19: 1094041	NM_002695:c.G94A:p.E32K	0.50
ALL-28	*KCNT2*	chr1: 196254866	NM_001287820:c.A2396C:p.N799T	0.51
ALL-28	*VOPP1*	chr7: 55605210	NM_001284284:c.C11T:p.T4M	0.51
ALL-28	*ARID1A*	chr1: 27023581	NM_006015:c.C687G:p.Y229X	0.51
ALL-28	*NOTCH1*	chr9: 139399344	NM_017617:c.T4799C:p.L1600P	0.52
ALL-28	*NOTCH1*	chr9: 139390791	NM_017617:c.C7400A:p.S2467X	0.53
ALL-30	*TBCEL*	chr11: 120957510	NM_001130047:c.A980T:p.Y327F	0.10
ALL-30	*CDH16*	chr16: 66948189	NM_001204746:c.C419G:p.T140S	0.10
ALL-30	*GTF2B*	chr1: 89318987	NM_001514:c.A860G:p.Q287R	0.11
ALL-30	*CA12*	chr15: 63632638	NM_001293642:c.A416G:p.E139G	0.12
ALL-30	*RBFOX1*	chr16: 7568326	NM_145891:c.G265A:p.A89T	0.13
ALL-30	*SYNJ1*	chr21: 34045832	NM_001160306:c.A1529G:p.K510R	0.13
ALL-30	*ZNF843*	chr16: 31447164	NM_001136509:c.G1007A:p.W336X	0.14
ALL-30	*GLIS3*	chr9: 4118658	NM_152629:c.T355C:p.F119L	0.15
ALL-30	*UBAC2*	chr13: 99853188	NM_001144072:c.G26C:p.G9A	0.15
ALL-30	*STAT5B*	chr17: 40359729	NM_012448:c.A1924C:p.N642H	0.15
ALL-30	*CHD4*	chr12: 6696651	NM_001297553:c.C3757T:p.R1253C	0.15
ALL-30	*GTF2IRD1*	chr7: 73944204	NM_001199207:c.C1327T:p.R443W	0.15
ALL-30	*WDR46*	chr6: 33255277	NM_001164267:c.T572C:p.L191P	0.16
ALL-30	*SCAF8*	chr6: 155143485	NM_014892:c.A1868G:p.K623R	0.17
ALL-30	*OR56A1*	chr11: 6048732	NM_001001917:c.A203C:p.Y68S	0.18
ALL-30	*TTC28*	chr22: 28379198	NM_001145418:c.A6457G:p.S2153G	0.18
ALL-30	*PCDHB10*	chr5: 140572561	NM_018930:c.A436G:p.T146A	0.18
ALL-30	*DCST1*	chr1: 155014051	NM_001143687:c.A635G:p.Q212R	0.19
ALL-30	*SLCO6A1*	chr5: 101755603	NM_001289004:c.A1213T:p.I405L	0.19
ALL-30	*DSC2*	chr18: 28672165	NM_004949:c.A253G:p.I85V	0.19

ALL-30	*GRIK3*	chr1: 37325498	NM_000831:c.C907T:p.R303W	0.20
ALL-30	*ADAM22*	chr7: 87792403	NM_004194:c.A1984C:p.M662L	0.20
ALL-30	*MED12*	chrX: 70344958	NM_005120:c.C2188T:p.R730X	0.46
ALL-31	*ALPI*	chr2: 233321919	NM_001631c.T535C:p.Y179H	0.41
ALL-31	*TFR2*	chr7: 100218641	NM_001206855:c.C1732T:p.R578W	0.17
ALL-31	*DGKI*	chr7: 137092669	NM_004717:c.G2896A:p.A966T	0.24
ALL-31	*PTPRN2*	chr7: 157903560	:NM_001308267:c.G1490A:p.R497H	0.46
ALL-31	*ZNF462*	chr9: 109686953	NM_021224:c.C760T:p.R254C	0.18
ALL-31	*FUOM*	chr10: 135169275	NM_001301827:c.G296A:p.R99Q	0.40
ALL-31	*MRGPRX3*	chr11: 18159273	NM_054031:c.C524T:p.T175M	0.45
ALL-31	*SLC6A4*	chr17: 28536197	NM_001045:c.G1513A:p.A505T	0.35
ALL-31	*STAT5B*	chr17: 40359729	NM_012448:c.A1924C:p.N642H	0.39
ALL-31	*ZNF812*	chr19: 9801637	NM_001199814:c.A542G:p.D181G	0.48
ALL-31	*DNM2*	chr19: 10906762	NM_001005360:c.G1222C:p.A408P	0.43
ALL-31	*DIDO1*	chr20: 61526496	NM_001193369:c.C2236T:p.R746C	0.49

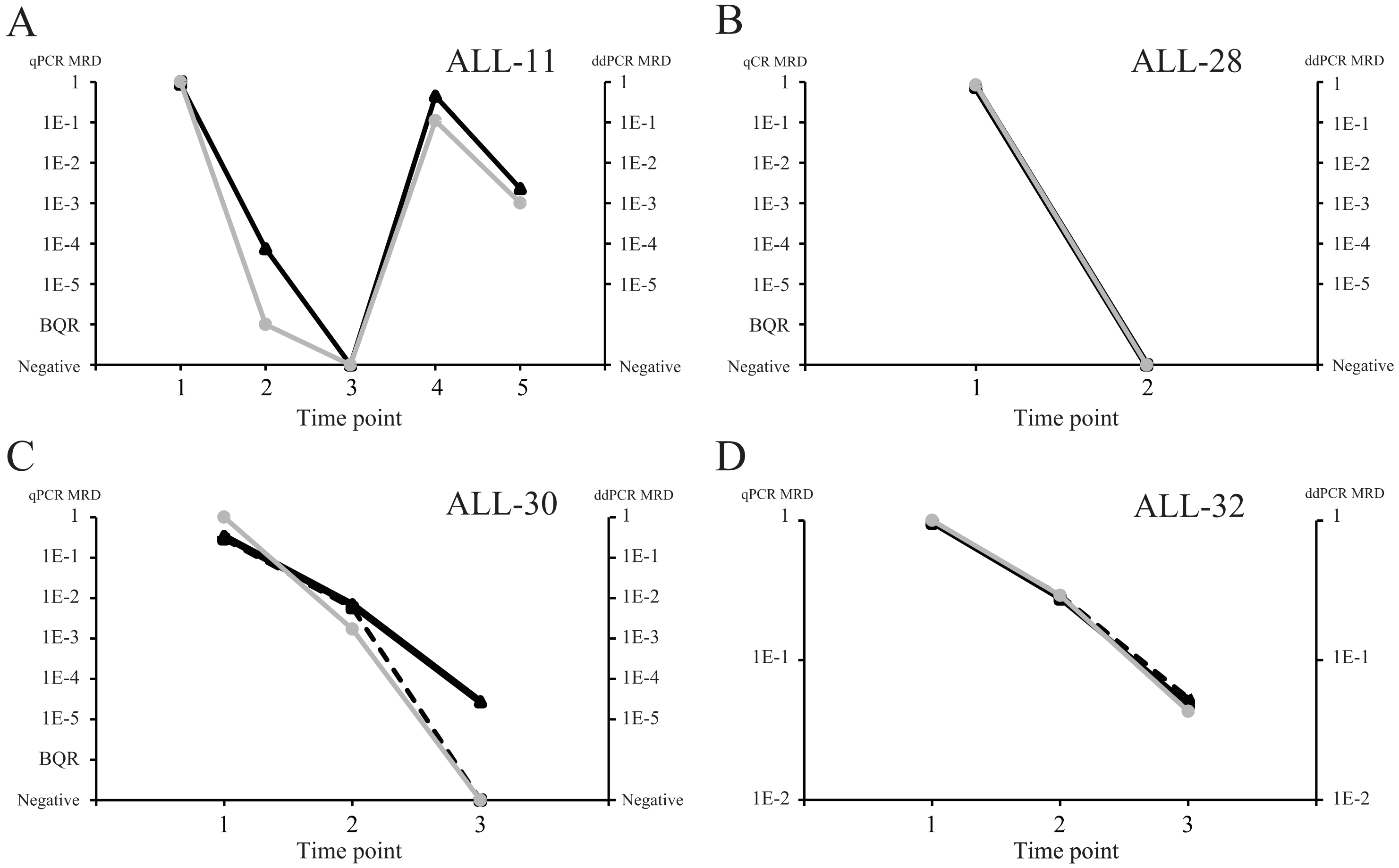

Figure S1. Results of MRD detection discordances in the follow-up samples of T-ALL patients Comparison of MRD level evaluated by qPCR (gray circle caps with lines) and ddPCR (black square caps with dashed lines or black triangle caps with straight lines). Time point 1 indicates the onset of disease. A, B: One primer/probe set for ddPCR was used in each case. C, D: Two different primer/probe sets for ddPCR were used in each case. ddPCR, droplet digital PCR; MRD, minimal residual disease; qPCR, real-time quantitative PCR; BQR, below the quantitative range

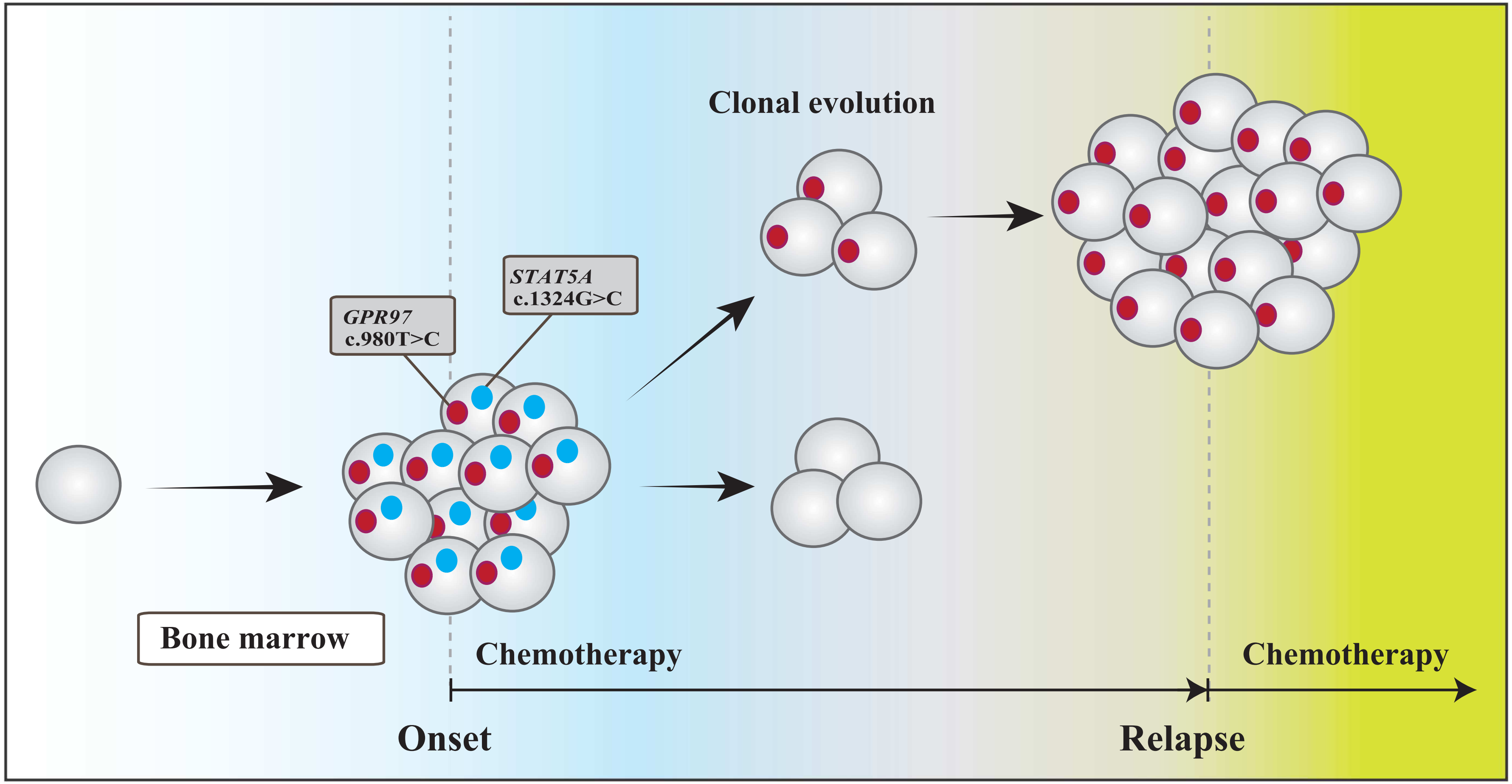

Figure S2. Schematic diagram of clonal evolution estimated from MRD analysis

STAT5A c.1324G>C targeted by ddPCR primer/probe set1 and *GPR97* c.980T>C targeted by ddPCR primer/probe set2 were common to the malignant cells at diagnosis, but only *GPR97* c.980T>C was detected in the relapsed clone. The occurence of clonal evolution in the tumor cells was estimated.
